# The Diagnostic Value of Procalcitonin in Patients with Severe Acute Pancreatitis: A Meta-Analysis

**DOI:** 10.5152/tjg.2022.22098

**Published:** 2022-09-01

**Authors:** Lipeng Chen, Jianbo Jiang

**Affiliations:** 1 Department of Gastroenterology, Zhejiang Hospital, Hangzhou, Zhejiang, China

**Keywords:** Diagnosis, meta-analysis, procalcitonin, severe acute pancreatitis

## Abstract

The role of procalcitonin in diagnosing severe acute pancreatitis has not been clearly assessed. This meta-analysis aims to evaluate the overall diagnostic accuracy of procalcitonin as a biomarker for severe acute pancreatitis. Medline (via PubMed), Embase, Web of Science, Cochrane Library, China National Knowledge Infrastructure, and China WanFang Data were searched systematically for prospective studies reporting procalcitonin as a diagnostic marker of severe acute pancreatitis before August 31, 2021. Sensitivity, specificity, and other measures of the accuracy of procalcitonin in the diagnosis of severe acute pancreatitis were pooled by Stata 15.0 software. Heterogeneity was evaluated by *I*^2^ test, and the quality of included studies was evaluated by using the Quality Assessment of Diagnostic Accuracy Studies-2 system. Further, the sources of heterogeneity were verified using meta-regression and subgroup analysis, and the publication bias was evaluated by the Deeks’ funnel plot. A total of 18 studies meeting the inclusion criteria were included, containing 1764 patients. The pooled sensitivity, specificity, positive likelihood ratio, negative likelihood ratio, diagnostic odds ratio, and area under the receiver operating characteristic curve of procalcitonin for diagnosing severe acute pancreatitis were as follows: 0.80 (95% CI: 0.73-0.86), 0.84 (95% CI: 0.78-0.88), 4.95 (95% CI: 3.46-7.09), 0.23 (95% CI: 0.16-0.34), 21.26 (95% CI: 11.09-40.74), 0.89 (95% CI: 0.86-0.92). Also, *P* > .05 suggested no significant publication bias. Current evidence indicates that procalcitonin has good sensitivity and diagnostic accuracy for severe acute pancreatitis. However, the findings should be carefully used as routine evidence in diagnosing patients with severe acute pancreatitis alone because of the limited number of included studies and high heterogeneity.

**Main Points**

- This is an updated systematic review of the diagnostic value of procalcitonin in severe acute pancreatitis (SAP).
- Procalcitonin has high sensitivity and diagnostic accuracy for SAP.
- There was no significant publication bias.
- Sensitivity analysis suggested that the results of this study were stable.

## Introduction

Acute pancreatitis (AP) is a common acute gastrointestinal inflammatory disease in the emergency department, indicated by abdominal pain radiating to the lower back, abdominal distension, nausea, or vomiting. It can even develop necrosis of the pancreas and surrounding tissues, accompanied by changes in serum enzymes such as amylase and lipase.^1^ As a digestive tract emergency expected to be hospitalized, AP has regional variations in its worldwide incidence (4.9-73.4 per 100 000 patients), and generally, its incidence has been on the rise in recent years.^2^ According to the revised Atlanta classification, AP can be classified into mild AP (MAP), moderately severe AP, and severe AP (SAP).^3^ Although the majority of patients with AP have mild clinical symptoms, about 20% of patients develop SAP, with a mortality rate as high as 15% or more.^4^ As a result of recent advances in treatment, the success rate of SAP resuscitation has increased markedly.^5^ However, in order to achieve a significant reduction in mortality of SAP, there is an urgent need for simple, fast, and sensitive predictors to diagnose patients at admission.

Currently, the most widely used clinical diagnostic scoring systems for predicting SAP are the Ranson score and the Harmless Acute Pancreatitis Score, Acute Physiology and Chronic Health Evaluation-II (APACHE-II), and Beside Index for Severity in Acute Pancreatitis (BISAP).^6^ Unfortunately, they have only moderate diagnostic accuracy. In addition, Ranson score and APACHE-II score are with the disadvantage of computational complexity. Some quick and inexpensive independent diagnostic markers, such as C-reactive protein (CRP) and hematocrit, also fail to be promoted clinically due to their low accuracy.^7^

Procalcitonin (PCT) was discovered in 1984^8^ and since then its biological characteristics and clinical application were widely concerned; its increased serum level in septic patients was first reported in 1993. Procalcitonin is usually undetectable in healthy control but shows changes in response to sepsis and infections.^9^ Procalcitonin is rarely released into the blood, so its concentration in the serum of healthy people is very stable (<0.1 ng/mL).^10^ Pathologically, PCT has pathogen-associated and damage-associated molecular patterns. During the infection-induced systemic inflammatory response, PCT is widespread released into the blood from the cells of the thyroid, lung, liver, pancreas, colon, and other organs.^11^ The use of PCT, a useful biological index, contributes to differentiating bacterial infectious diseases,^12^ especially with a high value in the diagnosis of sepsis.^13,14^ Previous reports supplied some evidence for PCT in the diagnosis of SAP, infected pancreatic necrosis (IPN), and death.^15,16^

In the past few decades, new progress has been made one after another, urgently demanding a systematic and in-depth review to answer whether PCT can serve as a diagnostic factor for SAP. This study aims to conduct a meta-analysis to investigate the role of PCT level in the accurate prediction of SAP. We have provided a scientific basis for the diagnosis of SAP and improved its treatment.

## MATERIALS AND Methods

### Search Strategy

Two authors independently searched Medline (via PubMed), Embase, Web of Science, Cochrane Library, and 2 Chinese databases such as China National Knowledge Infrastructure and China WanFang Data to identify relevant studies. Those published in any languages before August 31, 2021, reported the performance of PCT in the diagnosis of SAP were target articles. The present systematic review and meta-analysis were written according to the Preferred Reporting Items for Systematic Reviews and Meta-Analyses guidelines.^17^ The search terms were (“procalcitonin” OR “PCT”), AND (“severe acute pancreatitis” OR “SAP”) OR (“diagnosis” OR “sensitivity”). More articles were obtained from the references of the identified articles.

### Inclusion and Exclusion Criteria

Inclusion criteria were as follows: (1) studies addressing PCT for diagnosing SAP patients; (2) all patients with SAP or MAP diagnosed by the conventional “gold standard”; (3) studies directly or indirectly providing true positive (TP), false positive (FP), false negative (FN), true negative (TN), sensitivity (SEN) and specificity (SPE) of PCT; (4) only blood sample from human examined were included.

Exclusion criteria were as follows: (1) literature reviews, case reports, systematic reviews, conference abstracts, letters, animal or laboratory studies; (2) the data were duplicated or overlapping; (3) studies were not based on human subjects; (4) studies without diagnostic data of TP, FP, FN, TN, SEN, and SPE; (5) studies with specimens fewer than 10 patients; (6) studies focused on sterile versus infected necrotizing pancreatitis (INAP) patients.

### Data Extraction and Quality Assessment

The final included articles were assessed independently by 2 authors. The data extracted from the reports included author(s), publication year, country, detection methods, number of patients and controls, sample size, detection time, cut-off value, SEN, SPE, and methodological quality. The methodological quality of the included studies was evaluated by the Quality Assessment of Diagnostic Accuracy Studies (QUADAS)-2 tool, with each item scored as “yes,” “unclear,” or “no.” ^18^ The rating of “yes,” “unclear,” and “no” means the risk of bias from low to high. Any controversial questions between the 2 authors were resolved through discussion. Figures were all with 95% CIs, and *P* < .05 was considered statistically significant.

### Statistical Analyses

All statistical analyses were performed using STATA software version 15 (Stata Corporation, College Station, Tex, USA). The following measures of diagnostic accuracy were computed by the bivariate mixed-effects model: SEN, SPE, positive likelihood ratio (PLR), negative likelihood ratio (NLR), area under the curve (AUC), and diagnostic odds ratio (DOR). The *I* ^2^ test was used to detect statistical heterogeneity across the studies. In case of significant heterogeneity, meta-regression analysis and subgroup analysis were carried out for detecting the source of heterogeneity. Publication bias was evaluated via Deeks’ funnel plot. The threshold effect is a major source of heterogeneity in meta-analyses of diagnostic tests^19^; Spearman’s correlation coefficient and the typical “shoulder arm shape” in the summary receiver-operating characteristic (SROC) curve were used to estimate the threshold effect.

## Results

### Study Selection and Study Characteristics

**Figure 1.**
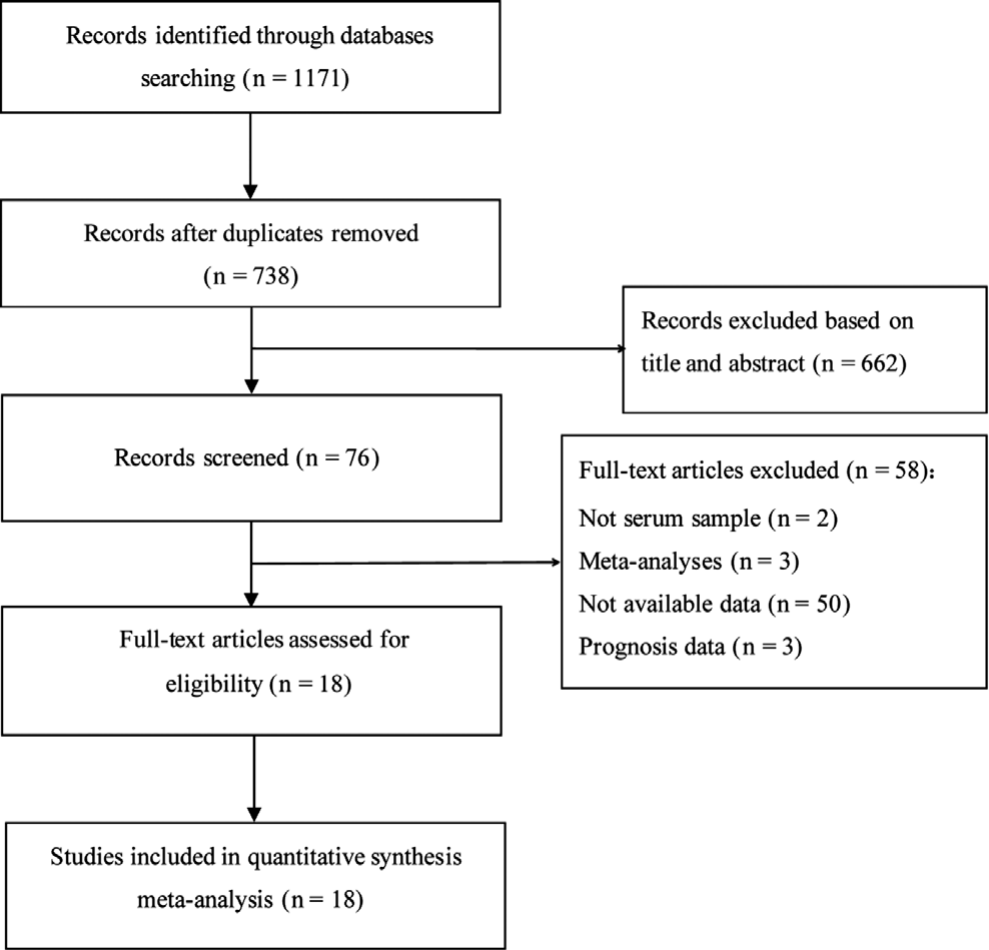
Flow diagram of the selection process for studies included in this meta-analysis.

A total of 18 articles were finally included in the meta-analysis (Figure 1). Specifically, 1171 articles were searched by the search strategy initially, followed by the exclusion of 433 duplicate articles. Then 662 articles, through reading their titles and abstract, failed to meet the inclusion criteria. After reviewing the full manuscripts of the remaining articles, 18 articles met the inclusion criteria with 667 SAP patients and 1097 MAP patients for the control group.^15,20-36^ Countries of publication were diverse (Italy, Finland, UK, Denmark, Turkey, Korea, Serbia, India, Sweden, Nepal, China). Except for 3 articles in Chinese,^29,31,33^ the rest were in English. The other detailed characteristics of included studies are shown in Table 1.

### Quality of Reports

**Figure 2.**
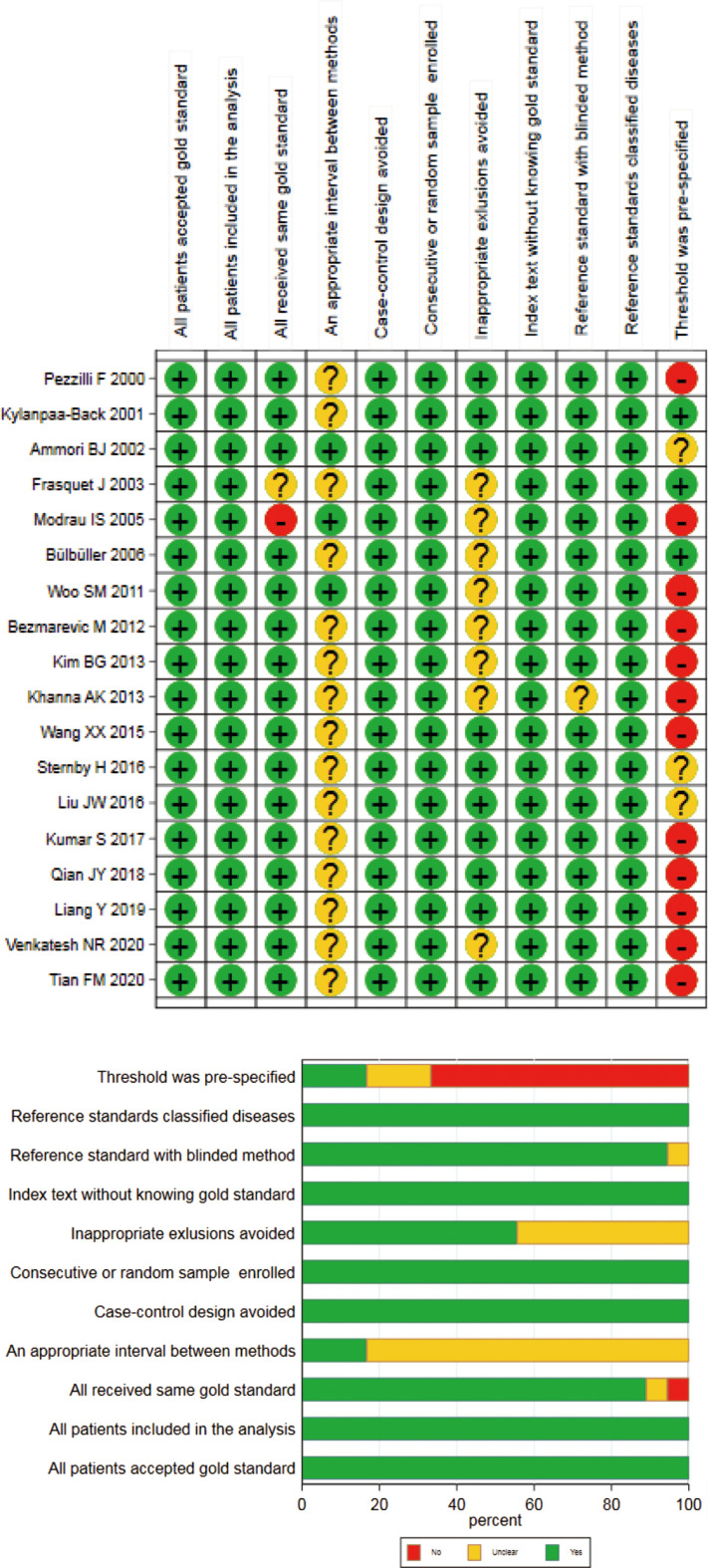
Quality analysis of included studies by Quality Assessment of Diagnostic Accuracy Studies-2 tool. (A) Risk of bias summary: risk of bias item for all studies. “+”: low risk of bias; “?”: unclear risk of bias; “-”: high risk of bias. (B) Risk of bias graph: risk of bias item presented as percentages among all studies.

The output of the quality analysis of included studies by the QUADAS-2 tool is presented in Figure 2. Cut-off values of 12 studies were calculated by SEN and SPE instead of preset threshold, which means “high risk” in the index test domain. Eight studies were “unclear” in the patient selection domain due to inappropriate exclusions, and 5 studies were “unclear” in the flow and timing domain because of an inappropriate interval between index tests. All studies had “low risk” in the reference standard domain. However, there were biases in the included studies.

### Diagnostic Accuracy and Heterogeneity Evaluation

**Figure 3.**
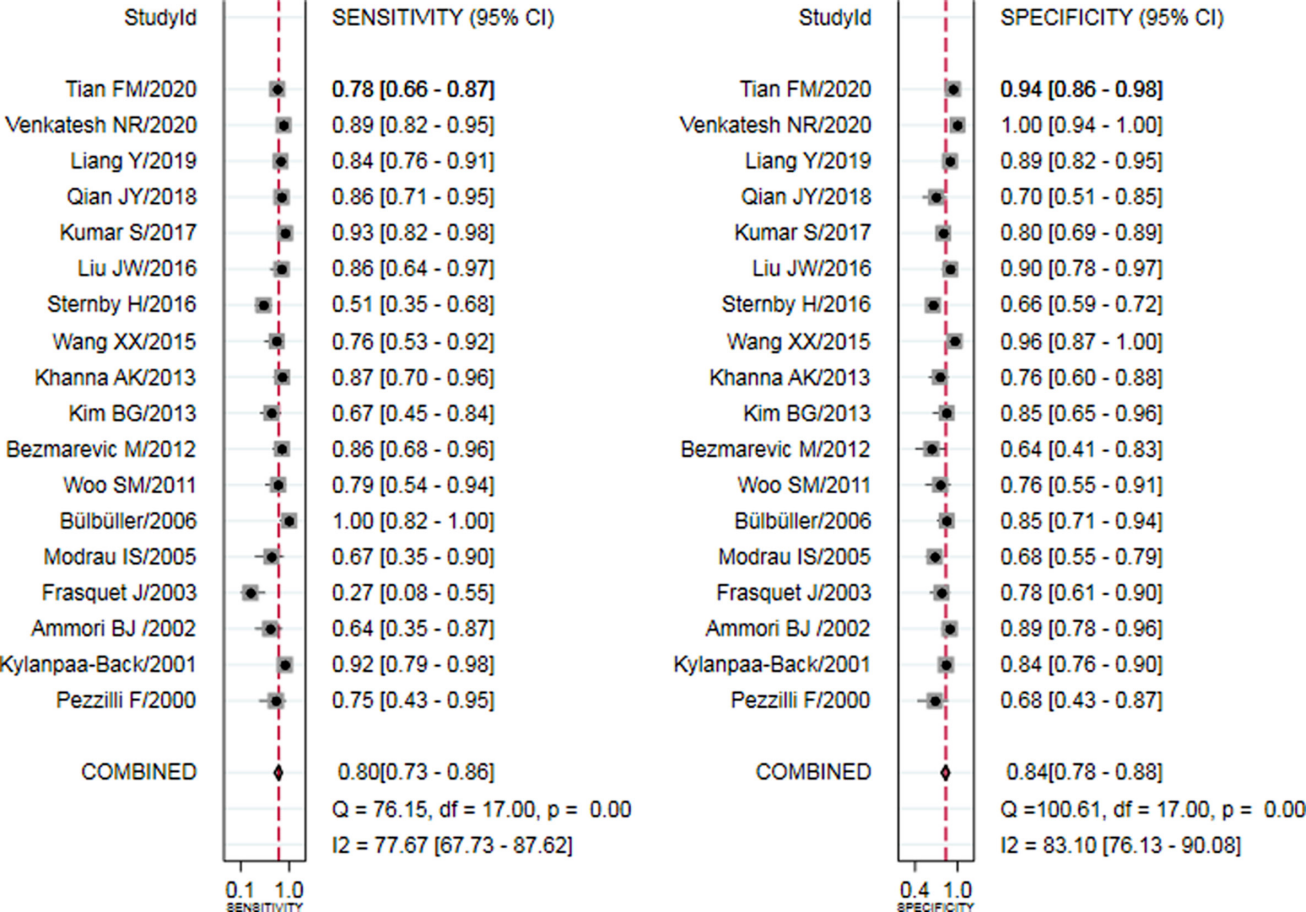
Sensitivity and specificity of procalcitonin in the diagnosis of SAP. SAP, severe acute pancreatitis.

**Figure 4.**
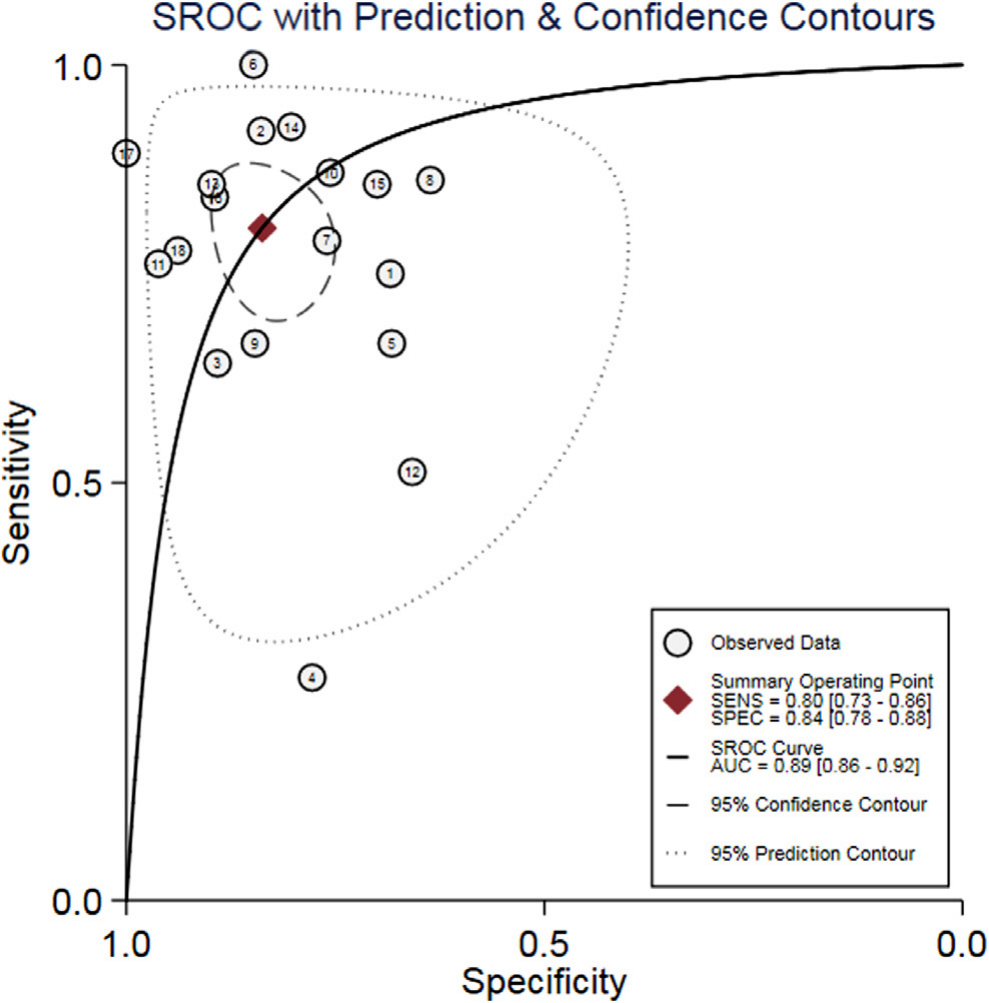
Summary of receiver operating characteristic curve for the accuracy of procalcitonin in the diagnosis of SAP. SAP, severe acute pancreatitis.

**Figure 5.**
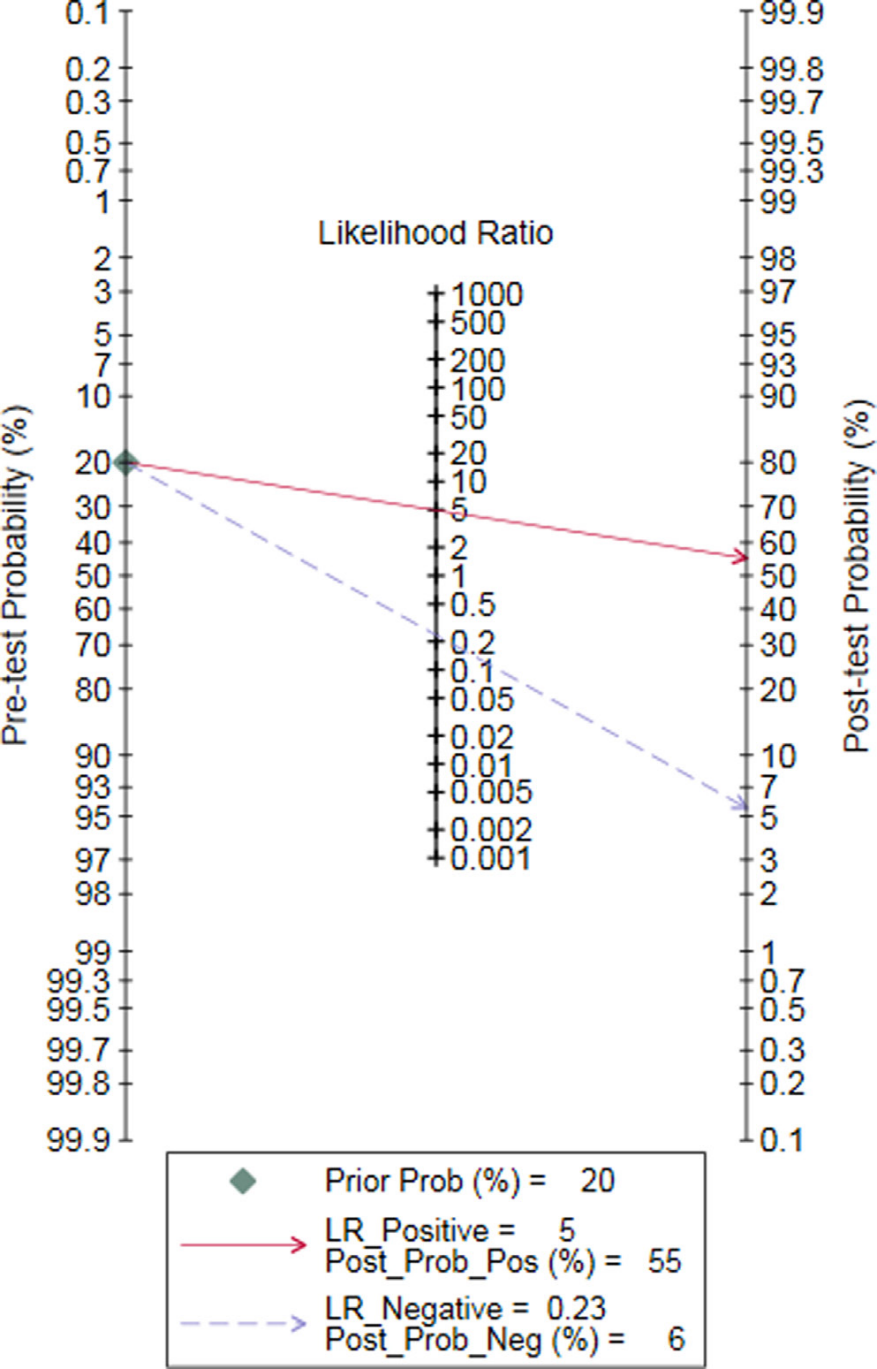
Fagan’s nomogram of procalcitonin in the diagnosis of SAP. SAP, severe acute pancreatitis.

Spearman correlation coefficient between the log of SEN and the log of 1 − SPE (−0.0883 with *P* = .7360) and no typical “shoulder arm shape” of SROC suggested no statistically significant difference in the threshold effect. The pooled SEN and SPE of blood PCT were 0.80 (95% CI: 0.73-0.86), 0.84 (95% CI: 0.78-0.88), respectively (Figure 3). The DOR was 21.26 (95% CI: 11.09-40.74), with heterogeneity between the studies (*I* ^2^ = 100%). The pooled PLR and NLR of PCT diagnostic accuracy were 4.95 (95% CI: 3.46-7.09) and 0.23 (95% CI: 0.16-0.34), respectively (Table 2). The AUC of PCT was 0.89 (95% CI: 0.86-0.92, Figure 4). According to Fagan’s Nomogram test, when the pretest probability was 20%, the post-test probability of PLR was 55% with PLR 5% while the post-test probability of NLR was 6% with NLR 0.2 (Figure 5).

### Meta-Regression Analysis and Subgroup Analysis

Ethnicity (Asian or Caucasian), detection method (BRAHMS immuno-luminometric assay (BIA) or others), sample size (≤75 or >75 patients), detection time (on admission or 24 hours), cut-off value (≤1 or > 1 ng/mL) were included in the regression model for detecting the source of heterogeneity. The degree of heterogeneity among studies was statistically significant in the following aspects: detection method (BIA or not) (*χ*^2^ = 20.51, *P* < .001, *I*^2^ = 90% [95% CI: 81-100%]), cut-off value (≤1 or > 1 ng/mL) (*χ*^2^ = 23.45, *P* < .001, *I*^2^ = 91% [95% CI: 83-100%]), detection time (on admission or 24 hours) (*χ*^2^ = 35.88, *P* < .001, *I*^2^ = 94% [95% CI: 90-99%]). The results are shown in Supplementary Table 1.

Further subgroup analysis took into consideration ethnicity, detection method, sample size, detection time, and cut-off value. By adjusting the detection method and sample size, the analysis revealed statistically significant differences in SPE between the BIA group and other methods group (0.79 vs 0.86, *P* < .001). In the meantime, there were mild differences in SEN among all different subgroups but not statistically significant (Supplementary Table 1).

### Sensitivity Analysis

**Figure 6.**
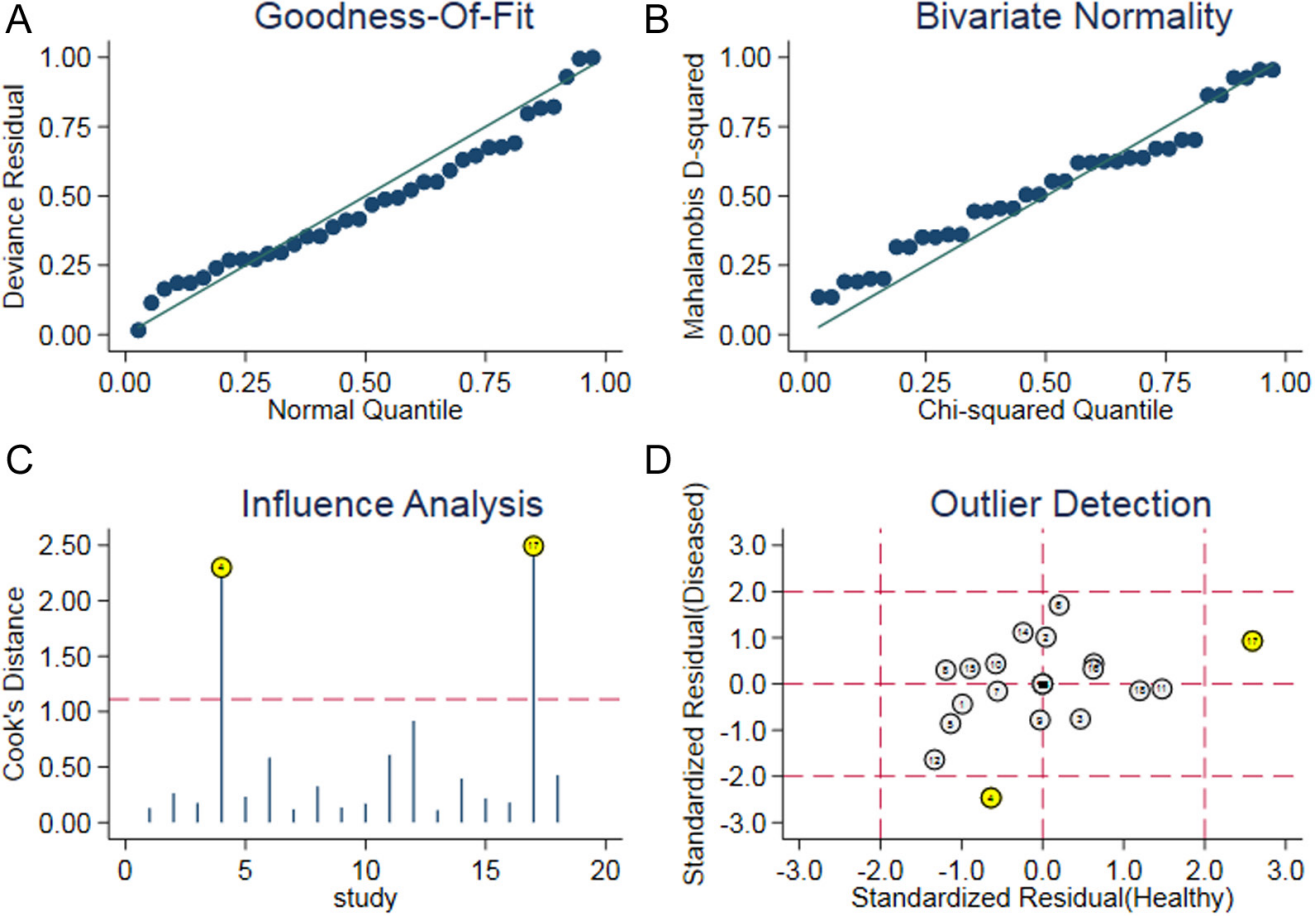
Sensitivity analysis of procalcitonin in the diagnosis of SAP. SAP, severe acute pancreatitis.

The SEN analysis of PCT as a predictor of SAP is displayed in Figure 6. Goodness of fit and bivariate normality showed the data were fitting well, suggesting the bivariate mixed-effect model was suitable for analysis (Figure 6A, B). Two weighted studies were found by impact analysis (Figure 6C). Outlier detection detected the same 2 abnormal studies (Figure 6D). After removing them, SEN increased slightly, while SPE, PLR, NLP, and DOR decreased slightly, and AUC had no change (Table 2, column 3). It was suggested that the results of this study were stable.

### Publication Bias

**Figure 7.**
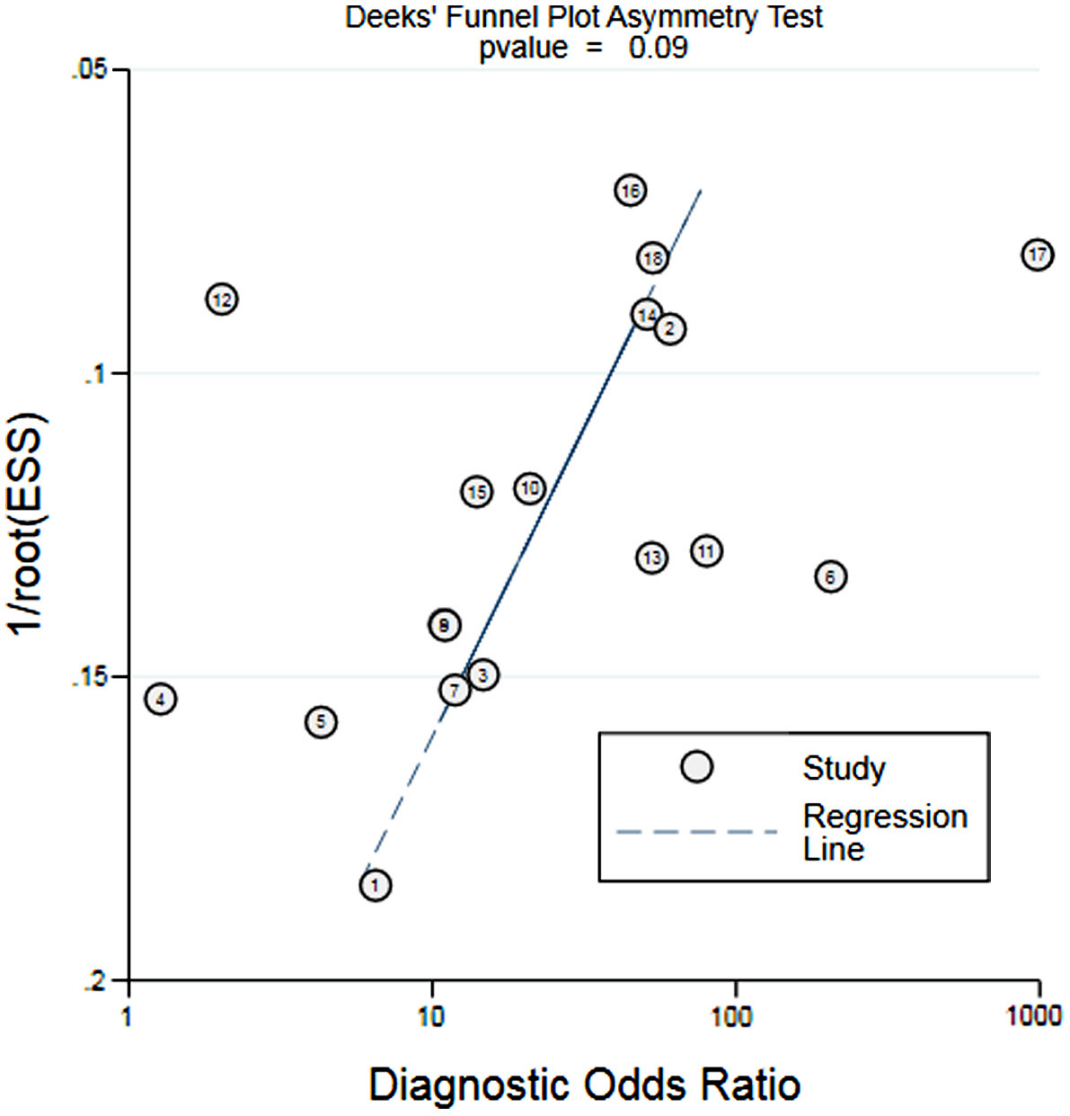
Result of publication bias by Deeks’ funnel plot asymmetry test.

The Deeks’ funnel plot asymmetry test of the included studies suggested no significant publication bias (Figure 7, *P* = .09).

## Discussion

Acute pancreatitis is a common acute gastrointestinal disease in surgical hospitalization. Its progression is rapid, and the mortality reaches 50% if patients suffer from secondary infections.^36^ Severe acute pancreatitis can lead to local complications such as pancreatic abscess, peripancreatic effusion, peripancreatic necrosis, and pancreatic pseudocyst. In addition, patients with SAP can have 1 or more organ dysfunction or even failure at an early stage, with the lungs and kidneys as the most commonly affected organs.^37^ Put simply, rapid progress of SAP requires special clinical attention, and timely and accurate assessment of the severity is of paramount importance for its treatment and prognosis. However, the usually used scoring systems such as APACHE-II, BISAP, and Systemic Inflammatory Response Syndrome score in clinical practice are complicated and not precise enough,^28,38^ leading to the failure of their clinical application and promotion. As such, it is urgent to find highly sensitive and specific, safe, fast, and easy-operated predictors for SAP. Procalcitonin has been found to be positively correlated with SAP and may therefore serve as a predictor for SAP,^23,24,39^ pancreatic necrosis, and organ failure.^15^ A recent meta-analysis including 8 studies with the pooled SEN, SPE, and AUC of PCT as a diagnostic marker for SAP was 0.73, 0.87, and 0.88, respectively, with the cut-off value of 0.5 ng/mL.^40^ Besides CRP, the gold standard for the diagnosis of SAP, PCT, and interleukin-6 have been reported in more studies as diagnostic factors and have been used in some hospitals, but not as a routine basis. Additionally, acute-phase proteins, cytokines, activation peptides of pancreatic proteases, antiproteases, adhesion molecules, and leukocyte-derived enzymes also present promising results but have not been implemented due to the drawbacks of low accuracy, high cost, or cumbersome operation.^41^ The exploring of new diagnostic molecules has never stopped.

In this study, we searched the studies that reported blood PCT as a predictor for SAP and pooled all data to confirm it was a good marker for predicting SAP. The pooled SEN and SPE of blood PCT in the diagnosis of SAP were 0.84 and 0.81, respectively. The pooled PLR indicated that SAP patients were 4.95 times to be diagnosed as positive than to be misjudged, while the pooled NLR indicated that misjudgment as negative of SAP patients were 23% of being correctly diagnosed as negative, revealing high diagnostic value. The DOR value was 21.26, and the AUC was 0.89, indicating that the accuracy of blood PCT in the diagnosis of SAP was good. Further, we explored the source of heterogeneity through meta-regression analysis and found cut-off value, detection method, and timing may be answers. According to the SEN analysis, we were sure of the stability of this study. Previous meta-analyses on the diagnostic accuracy of PCT for SAP had contradictory but comparable results. Shafiq et al^42^ first reported meta-analysis with 4 studies about assessing the severity of SAP, but they concluded that PCT cannot be considered as a good marker. Then the following meta-analysis including 9 studies with types of MAP versus SAP demonstrated that serum PCT for predicting sterile and INAP had a moderate SEN but higher accuracy in the latter.^43^ A recent systematic review with 24 studies assessed the serum PCT as an accurate predictor of SAP and IPN.^40^

However, this study has the following limitations: (1) There was heterogeneity among the studies in calculating the overall effects. The results of this study are expected to be confirmed by large-scale prospective clinical studies. (2) This study only discussed the potential value of PCT in the diagnosis of SAP on blood samples. Plasma level of PCT was detected in 1 study which had a good diagnostic effect,^44^ while the remaining studies were serum sample studies. No subgroup analysis of serum and plasma was performed in this study due to the finite number of studies with plasma samples. (3) The multi-factor combination in the diagnosis of SAP was not analyzed in this study. (4) The correlation between blood PCT and diagnosis of organ failure, infection, death in patients with SAP was not analyzed. (5) Published studies had usually positive results, which might lead to sort of bias. The defects mentioned above can guide future research. Put simply, more and deeper researches are necessary to confirm our conclusion.

## Conclusion

This meta-analysis indicates that the diagnostic value of blood PCT is good in patients with SAP, but more studies with larger sample size are still needed to verify the findings. It is recommended to consider the blood PCT level as an auxiliary marker to scoring systems in the diagnosis of SAP.

## Figures and Tables

**Table 1.** Characteristics of Included 18 Studies

**First Author**	**Year**	**Country**	**Detection Method**	**MAP**	**SAP**	**Time of Blood Samples**	**Cut-off Value** **(ng/mL)**	**TP**	**FP**	**FN**	**TN**	**SEN**	**SPE**
Pezzilli^20^	2000	Italy	BIA	19	12	On admission	0.252 -0.255	9	6	3	13	0.768	0.693
Kylanpaa-Back^15^	2001	Finland	BIA	124	38	24 hours	0.5	35	20	3	104	0.92	0.84
Ammori^21^	2002	United Kingdom	BIA	55	14	On admission	0.5	9	6	5	49	0.67	0.89
Frasquet^22^	2003	Spain	BIA	36	15	24 hours	0.5	4	8	11	28	0.267	0.777
Modrau^23^	2005	Denmark	BIA	63	12	48 hours	0.5	8	20	4	43	0.67	0.68
Bülbüller^24^	2006	Turkey	BIA	46	19	On admission	0.5	19	7	0	39	1	0.84
Woo^25^	2011	Korea	BIA	25	19	24 hours	1.77	15	6	4	19	78.9	76
Bezmarevic^26^	2012	Serbia	BIA	22	29	24 hours	0.25	25	8	4	14	0.86	0.63
Kim^27^	2013	Korea	BIA	26	24	On admission	3.29	16	4	8	22	66.67	84.62
Khanna^28^	2013	India	BIA	41	31	24 hours	0.5	27	10	4	31	86.4	75
Wang^29^	2015	China	ECL	52	21	24 hours	NR	16	2	5	50	0.762	0.961
Sternby^30^	2016	Sweden	ELISA	193	39	On admission	0.35	20	66	19	127	0.52	0.66
Liu^31^	2016	China	ELISA	49	21	NR	NR	18	5	3	44	0.864	0.897
Kumar^32^	2017	Nepal	BIA	71	54	24 hours	0.9	50	14	4	57	0.926	0.803
Qian^33^	2018	China	ELISA	30	42	24 hours	3.25	36	9	6	21	0.867	0.694
Liang^34^	2019	China	ELISA	104	101	On admission	1.8	85	11	16	93	84.62	89.11
Venkatesh^35^	2020	India	NR	60	104	On admission	1.5	93	0	11	60	89.66	100
Tian^36^	2020	China	Immunofluorescence	81	72	6 hours	2.29	56	5	16	76	0.778	0.94

TP, true positive; FP, false positive; TN, true negative; FN, false negative; MAP, mild acute pancreatitis; SAP, severe acute pancreatitis; BIA, BRAHMS immuno-luminometric assay; ECL, electrochemiluminescence; ELISA, enzyme-linked immunosorbent assay; NR, no report; SEN, sensitivity; SPE, specificity.

**Table 2.** Diagnostic Performance of PCT After Removing the 2 Weight Researches

Effect Size	18 Studies	16 Studies (Removed Frasquet [22] and Venkatesh NR [35])
SEN	0.80 (95% CI:0.73-0.86)	0.82 (95% CI:0.75-0.87)
SPE	0.84 (95% CI:0.78-0.88)	0.82 (95% CI:0.77-0.87)
PLR	4.95 (95% CI:3.46-7.09)	4.6 (95% CI:3.4-6.2)
NLR	0.23 (95% CI:0.16-0.34)	0.22 (95% CI:0.16-0.31)
DOR	21.26 (95% CI:11.09-40.74)	21 (95% CI:12-36)
AUC	0.89 (95% CI:0.86-0.92)	0.89 (95% CI:0.86-0.91)

SEN, sensitivity; SPE, specificity; PLR, positive likelihood ratio; NLR, negative likelihood ratio; DOR, diagnostic odds ratio; AUC, area under the curve; PCT, procalcitonin.

**Supplementary Table 1.** Meta-Regression Analysis and Subgroup Analysis

Parameter	Category	Number of Studies	Subgroup Analysis			Meta-Regression Analysis			
SEN	*P*	SPE	*P*	LRTchi	*P*	*I* ^2^
Ethnicity	Asian	10	0.84 (95% CI: 0.77-0.91)	.38	0.88 (95% CI: 0.83-0.93)	.14	5.76	.06	65 (95% CI: 22-100)
Caucasian	8	0.74 (95% CI: 0.62-0.86)		0.77 (95% CI: 0.68-0.86)				
Detection method	BIA	11	0.81 (95% CI: 0.72-0.90)	.18	0.79 (95% CI: 0.72-0.85)	0	20.51	0	90 (95% CI: 81-100)
Other	6	0.79 (95% CI: 0.67-0.91)		0.86 (95% CI: 0.80-0.93)				
Sample size	≤75	8	0.82 (95% CI: 0.72-0.91)	.12	0.85 (95% CI: 0.77-0.92)	.02	0.15	.93	0 (95% CI: 0-100)
>75	10	0.79 (95% CI: 0.70-0.89)		0.83 (95% CI: 0.76-0.91)				
Detection time	On admission	7	0.79 (95% CI: 0.67-0.91)	.08	0.86 (95% CI: 0.79-0.93)	.13	35.88	0	94 (95% CI: 90-99)
24 hours	8	0.83 (95% CI: 0.73-0.93)		0.80 (95% CI: 0.71-0.89)				
Cut-off value	≤1	7	0.81 (95% CI: 0.70-0.92)	.19	0.87 (95% CI: 0.81-0.93)	.1	23.45	0	91 (95% CI: 83-100)
>1	9	0.80 (95% CI: 0.69-0.90)		0.78 (95% CI: 0.70-0.85)				

SEN, sensitivity; SPE, specificity; ELISA, enzyme-linked immunosorbent assay; BIA, BRAHMS immuno-luminometric assay.
